# 

**Published:** 1882-10

**Authors:** 


OCTOBER, 1882—CONTENTS. PAGE

ORIGINAL COMMUNICATIONS—Recent Discoveries of Fossil Horses. J. L. Wortman . . . 281
The Teeth of the Horse and their Diseases R. Jennings, V.S. . . . . . . . . . . . . . . . . . . 288
A Lesson in Comparative Zoology. Hubbard W. Mitchell, M.D. . . . . . . . . . . . . . . . . . 292
The Care of Cutaneous Diseases of the Elephant in Captivity. Dr. Max Schmidt . . . . . . . . . 301
Some Diseases of the Foot. Henry C. Slee . . . . . . . . . . . . . . . . . . . . . . . . . . . 303

EDITORIAL—Insanity in the Lower Animals—Virgil as a Veterinary Poet—Massage in Veterinary Therapeutics—The Test for Glanders by Inoculation—The Hypodermic Use of Morphine in the Colic of Horses—Slaughter-House Examinations—The Cure for Hydrophobia—Literary Notes—The Frequeney of Tuberculosis in Cows Slaughtered for Food—The Flesh and Milk of Tuberculous Cows . . . . . . . . . . . . . . . . . . . . . . . . . . . . . . . . . . . . . . . . 309 316

CASE DEPARTMENT—Diffuse Purulent Bronchiectasy in Colts, with Morbid Anatomy Notes—Hæmato-Pyo-Metra in a Bitch—Catarrhal Phthisis in a Llama, with a Fibro-Calcareous Tumor in the Perineal Region—Caries of a Molar Tooth of the Superior Maxilla, with Necrosis of the Jaws—Inversion of the Uterus . . . . . . . . . . . . . . . . . . . . . . . . . . . . . . . . . . 317–325

PROCEEDINGS OF SOCIETIES—Pathological Society of Philadelphia . . . . . . . . . . . . 326

OBITUARY . . . . . . . . . . . . . . . . . . . . . . . . . . . . . . . . . . . . . . . . . . . . 327

REVIEWS—Report of the Treasury Cattle Commission on the Lung Plague of Cattle, or Contagious Pleuro-Pneumonia—Books and Pamphlets received . . . . . . . . . . . . . . . . . . . . . . . 328–329

PROGRESS OF VETERINARY SCIENCE — Camel's Lung with Filariæ Sanguinis—A Blood Diet for the Herbivora—The Salmon Disease and its Lessons . . . . . . . . . . . . . . . . . . . 330–331

NEWS AND MISCELLANY—Feeding Horses—The Pulse of Different Domestic Animals—The Cow Population—Natural History and Education—Veterinary Honors—The Record of Casualties in the Conveyance of Live Stock by Sea—Veterinary Department at Harvard—Unknown Disease among Cattle—The Cattle Quarantine—A Horse with a Grievance—Bovine Post-mortem—Ought Diseased Meat to be used as Human Food?—Arab Horses—The Census of Live Stock—A Novel Cure for Hydrophobia—Trotters for New Zealand . . . . . . . . . . . . . . . . . . . . . . . 332–340

